# Super Tough PA6/PP/ABS/SEBS Blends Compatibilized by a Combination of Multi-Phase Compatibilizers

**DOI:** 10.3390/ma17215370

**Published:** 2024-11-02

**Authors:** Jianhui Yan, Cuifang Wang, Tongyu Zhang, Zijian Xiao, Xuming Xie

**Affiliations:** Key Laboratory of Advanced Materials (MOE), Department of Chemical Engineering, Tsinghua University, Beijing 100084, China

**Keywords:** polymer blends, compatibilization, multi-phase compatibilizer, superior mechanical properties, recycling of waste plastics

## Abstract

Development of multi-component blends to prepare high-performance polymer materials is still challenging, and is a key technology for mechanical recycling of waste plastics. However, a multi-phase compatibilizer is prerequisite to create high-performance multi-component blends. In this study, POE-*g*-(MAH-*co*-St) and SEBS-*g*-(MAH-*co*-St) compatibilizers are prepared via melt-grafting of maleic anhydride (MAH) and styrene (St) dual monomers to polyolefin elastomer (POE) and poly [styrene-b-(ethylene-co-butylene)-b-styrene] (SEBS), respectively. Subsequently, these compatibilizers are utilized to compatibilize the PA6/PP/ABS/SEBS quaternary blends through melt-blending. When POE-*g*-(MAH-*co*-St) and SEBS-*g*-(MAH-*co*-St) are added, respectively, both can promote the distribution of the dispersed phases, significantly reducing the dispersed phase size. When adding 10 wt% POE-*g*-(MAH-*co*-St) and 10 wt% SEBS-*g*-(MAH-*co*-St) together, compared to the non-compatibilized blend, the fracture strength, fracture elongation, and impact strength surprisingly increased by 106%, 593%, and 823%, respectively. It can be attributed to the hierarchical interfacial interactions which facilitate gradual energy dissipation from weak to strong interfaces, resulting in the improvement of mechanical properties. The synergistic effect of the enhanced phase interfacial interactions and toughening effect of elastomer compatibilizer achieved simultaneous growth in strength and toughness.

## 1. Introduction

Polymer blending has become one of the most convenient and effective methods for preparing high-performance new polymer materials in the past decades [1,2]. Also, it is a powerful and crucial technique for the recycling and upgrading of plastic waste [3,4]. Typically, the compatibility of binary polymer blends can be easily achieved by adding prefabricated block or graft copolymers with chemical affinity [5,6,7,8,9,10,11,12,13,14,15] or reactivity [16,17,18,19,20,21,22,23,24,25,26,27] to the two polymer components at interfaces. However, with ternary or multi-phase immiscible polymer blends, these strategies have limited effectiveness due to the multi-interface among polymer components and complex thermodynamic complications [28,29,30,31]. It is of great importance to study immiscible multi-component polymer blends to develop and prepare new materials with desired high-performance properties using favorable combinations of polymers. Meanwhile, with the growing concern for environmental pollution and sustainable development, the recycling of waste plastics has attracted wide attention [32,33,34,35]. Therefore, compatibilization of immiscible multi-component polymer blends is more and more becoming a crucial and powerful method for mechanical recycling of mixed waste polymers.

Over the past few decades, a series of compatibilizers have been applied to various incompatible blend systems. Recently, Eagan et al. [36] reported *i*PP-PE multiblock copolymers as isotactic polypropylene (*i*PP) and high-density polyethylene (HDPE) compatibilizers, with the reduce of average dispersed phase size from 2 μm to 1 μm and increasement of elongation at break from 10% to 500%. Nomura et al. [37] synthesized PET-PE multiblock polymers as compatibilizer additives in blends and as adhesion layer in multilayer films.

In addition, the simultaneously use of compatibilizers has also been reported. Lee et al. [38] used polypropylene graft maleic anhydride (PP-*g*-MAH) and polyethylene graft glycidyl methacrylate (PE-*g*-GMA) as a hybrid compatibilizer for PP/PLA blend which had better compatibilization effects compared to a single compatibilizer, with higher mechanical properties and smaller decrease in mechanical properties after acidic hydrolysis test. Wang et al. [39] designed a dual compatibilizer consisting of ethylene methyl acrylate-glycidyl methacrylate terpolymer (EMA-*co*-GMA) and maleic anhydride grafted polypropylene (PP-*g*-MAH) to compatibilize methyl vinyl silicone rubber/polypropylene (MVQ/PP) blends. The significant increase in interface thickness and reduction in MVQ dispersed phase size demonstrate the effective compatibilization effect. Aziz et al. [40] studied the effect of the combination of PP-g-MAH and SAM compatibilizers in the PP/PS blends, which produced a smaller dispersed phase size and higher impact strength compared to single compatibilizer system. Moreover, polyethylene graft maleic anhydride (PE-*g*-MAH) with a long backbone length and polybutadiene graft maleic anhydride (PB-*g*-MAH) with a short backbone length were introduced into immiscible low-density polyethylene/polyamide 6 (LDPE/PA6) blends by Ren et al. [41], enhancing the development of smooth and tightly linked interfaces, leading to the formation of a stable co-continuous morphology with a high degree of continuity in the blend comprising 30 wt% PA. However, most of the research about combination of compatibilizers is focused on binary blends, while it is still a challenge in ternary and multi-component blend systems.

In our previous works [42,43,44,45,46,47,48,49], the multi-phase compatibilizer PP-*g*-(MAH-*co*-St) and SEBS-*g*-(MAH-*co*-St) prepared by melt-grafting maleic anhydride (MAH) and styrene (St) dual monomers to PP were used to compatibilize the immiscible ternary PP/PA6/PS blends and quaternary PA6/PS/PP/SEBS blend. It shows very good compatibility in the blends with finer dispersion of dispersed phase and better interfacial adhesion, resulting the significant increase in mechanical properties. Based on our long-term work, the concept of “super composites” is proposed for multi-component blends. The hierarchical interfacial interactions among the components not only facilitate finer and organized dispersion but also facilitate gradual energy dissipation from weaker to stronger interfaces under strain, resulting in the mechanical properties improving exponentially, ultimately leading to the formation of “super composites”.

**Scheme 1:**
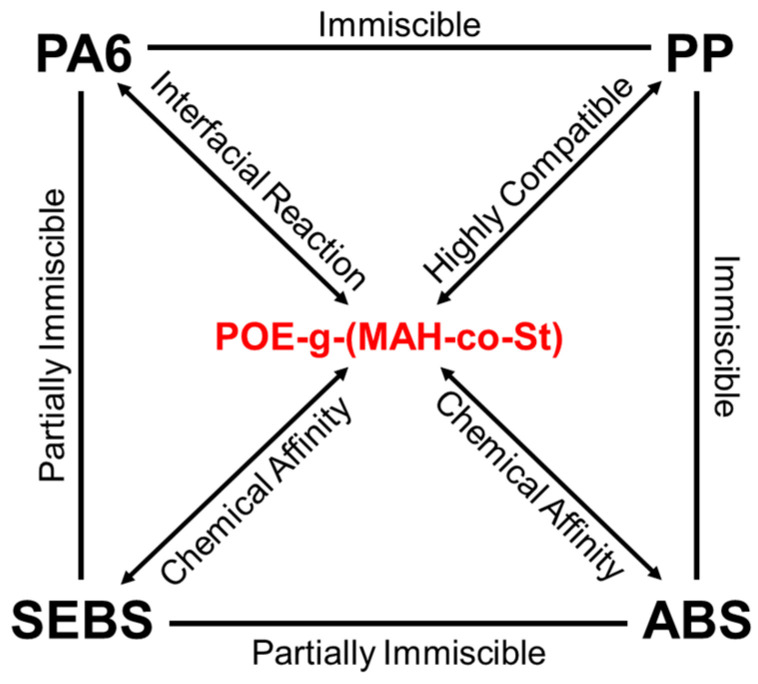
Schematic description of the compatibilization for PA6/PP+POE-*g*-(MAH-*co*-St)/ABS/SEBS quaternary blends.

In this work, two kinds of elastomer compatibilizers were prepared by melt-grafting MAH and St dual monomers to SEBS and polyolefin elastomer (POE, PP-PE copolymer) which were used as multi-phase compatibilizers for PA6/PP/ABS/SEBS quaternary blends to investigate the effect of the different affinities of each compatibilizer as a component in the blends on the morphology and mechanical properties of the blends. Taking POE-*g*-(MAH-*co*-St) as the example, the compatibilization strategy is described in Scheme 1. The anhydride group from grafted MAH has high reactivity with amino group in PA6 which can form the amide group. The styrene (St) block in graft chain of compatibilizer has better affinity with SEBS and ABS which also contains a large number of styrene blocks. Meanwhile, SEBS contains a polyolefin block consisting of ethylene and butylene segments which shows certain chemical affinity with polyolefin POE and PP. It is obvious that POE-*g*-(MAH-*co*-St) has better affinity with PP, and SEBS-*g*-(MAH-*co*-St) has higher chemical affinity with SEBS and ABS. This study aims to clarify the effects of the combination of different compatibilizers on the morphology, development, strengthening, and toughening of PA6/PP/ABS/SEBS blends.

## 2. Materials and Methods

### 2.1. Materials

PA6 (1013B) was from Ube Chemicals Co., Ltd. (Yamakuchi, Japan). PP (T30S) was supplied by Kunlun petrochemical Co., Ltd. (Daqing, China), with the melt flow index of 3.2 g·(10 min)^−1^ at 213 °C, 2.16 kg. ABS (8434A) was obtained from Liaoning Huajin Chemical Industrial Group Corporation (Panjin, China). POE (Versify-2300, ethylene-propylene copolymer) was supplied from Dow Chemical Company (Midland, MI, USA), with ethylene content of 12 wt% and the melt flow index of 2.0 g·(10 min)^−1^ at 213 °C. Poly [styrene-*b*-(ethylene-*co*-butylene)-*b*-styrene] (SEBS) Tuftec™ H1051 was supplied by Asahi Kasei Chemicals Corporation (Tokyo, Japan). Maleic anhydride (MAH) and styrene (St, AR) was purchased from Macklin Reagent (Shanghai, China). The initiator Dicumyl peroxide (DCP) was supplied by Bejing Xizhong Chemical Co., Ltd (Beijing, China). All reagents were used without further purification.

### 2.2. Preparation of Multi-Phase Compatibilizers

The multi-phase compatibilizers SEBS-*g*-(MAH-*co*-St) and POE-*g*-(MAH-*co*-St) were prepared by the multi-monomer melt-grafting method using a twin-screw extruder (D = 26 mm, L/D = 40) at 195 °C with a screw speed of 80 rpm. MAH, St, and DCP were premixed first with SEBS or POE pallets then added to twin-screw extruder. The concentration of MAH, St, and DCP were 3 wt%, 3 wt%, and 0.3 wt%, respectively, to SEBS or POE. The grafting ratios of MAH in SEBS-*g*-(MAH-*co*-St) and PP-*g*-(MAH-*co*-St) were 2.73 wt% and 1.55 wt% which was determined by a back titration procedure [50]. *g*-SEBS and *g*-POE are used to refer to SEBS-*g*-(MAH-*co*-St) and POE-*g*-(MAH-*co*-St), respectively.

### 2.3. Preparation of Quaternary Blends

PA6/(PP+*g*-POE)/ABS/SEBS (70/15/15/15), PA6/PP/ABS/(SEBS+*g*-SEBS) (70/15/15/15) and PA6/(PP+*g*-POE)/ABS/(SEBS+*g*-SEBS) (70/15/15/15) blends were prepared via melt-blending using a twin-screw extruder. For the PA6/PP/ABS/SEBS blend without compatibilizer, the weight amounts of PA6, PP, ABS, and SEBS are set to 70/15/15/15. In this case, weight amounts of PA6, PP, and ABS are counted as total amount with SEBS elastomer excluded. With increasing *g*-POE replacing PP, the total amount of PP and *g*-POE remain unchanged at 15 wt%. With *g*-SEBS replacing SEBS, the total amount of SEBS and g-SEBS remain unchanged at 15 wt%. The barrel temperature was 205–240 °C from the hopper to die with a screw rotation speed of 120 rpm. The content of each compatibilizer was set at 0 wt%, 5 wt%, 10 wt%, and 15 wt%, respectively. Then, the pelletized blends were injection molded into ASTM D638 tensile specimens and ASTM D256 notch impact specimens using injection modeling machine (M1200, Wuhan Qien Science & Technology Development Co., Ltd., Wuhan, China) for further mechanical tests.

### 2.4. Characterization

#### 2.4.1. ATR−FTIR Analysis

1 g of *g*-POE was dissolved in 100 mL of xylene at 135 °C for 2 h under reflux, and then excess isopropanol was added to precipitate the grafted material to remove small molecule monomers and un-grafted polymers. The precipitate was washed three times with isopropanol and dried at 60 °C under vacuum for 12 h. The attenuated total reflectance – Fourier transform infrared spectroscopy (ATR−FTIR) of purified *g*-POE, purified *g*-SEBS and virgin POE, virgin SEBS were collected on a IRTracer-100 (Shimadzu Corporation, Kyoto, Japan) with 32 scans at a resolution of 4 cm^−1^.

#### 2.4.2. SEM Observation

With a field emission scanning electron microscopy (JSM-7401, JOEL Ltd., Tokyo, Japan) set to a 3 kV accelerating voltage, the morphology of blends’ fractured surface was investigated. The samples were cryogenically fractured after being submerged in liquid nitrogen and the fractured surfaces were etched with *n*-heptane for 12 h at 35 °C to remove the SEBS phase in blends. The fractured surface was coated with platinum layer before SEM observation.

#### 2.4.3. Mechanical Tests

The uniaxial tensile test specimens with dimensions of 5 mm (width) × 35 mm (length) × 2 mm (thickness) were tested using Instron universal testing machine under a tensile speed of 50 mm/min according to ASTM D638 standard. For the Izod notched impact strength test, the rectangular specimens with dimensions of 10 mm (width) × 80 mm (length) × 4 mm (thickness) and a V-shaped notch with 45° angle and 2.0 mm depth were tested using Jinjian XJUD−5.5 impact tester with a pendulum of 5.5 J according to ASTM D256 standard. For each sample and mechanical test, at least five specimens were tested.

## 3. Results and Discussion

### 3.1. FTIR Spectroscopy Analysis of the Compatibilizers

**Figure 1:**
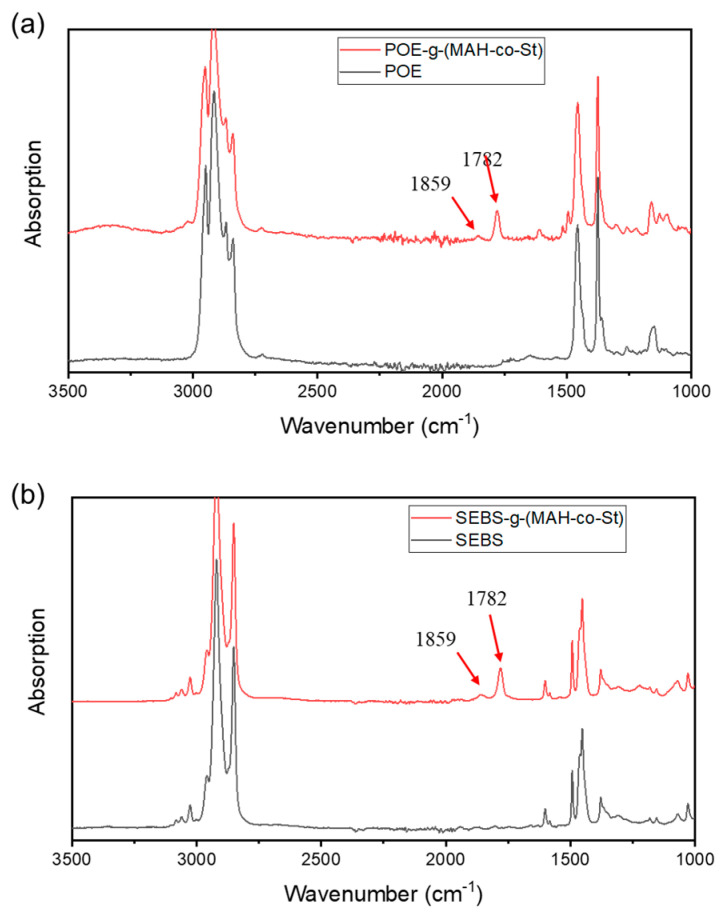
ATR−FTIR spectra of (**a**) virgin POE and POE-*g*-(MAH-*co*-St); (**b**) virgin SEBS and SEBS-*g*-(MAH-*co*-St).

The FTIR spectra of the prepared compatibilizers and virgin polymers are shown in Figure 1. Comparing with virgin POE or SEBS, new absorption bands at around 1860 and 1783 cm^−1^ in POE-*g*-(MAH-*co*-St) and SEBS-*g*-(MAH-*co*-St) were observed which can be assigned to symmetrical and unsymmetrical stretching modes of the C=O in anhydride [50,51]. Therefore, it indicates that POE and SEBS molecules have both grafted reactive MAH groups. The grafting ratios of MAH in *g*-POE and *g*-SEBS are 1.55 wt% and 2.06 wt%, determined by a back titration [50].

### 3.2. Morphology

In this study, POE-*g*-(MAH-*co*-St) and SEBS-*g*-(MAH-*co*-St) were used as compatibilizers to be blended with a PA6/PP/ABS/SEBS quaternary blend. The content of each compatibilizer was changed to investigate the effect on the morphology evolution and mechanical properties.

**Figure 2:**
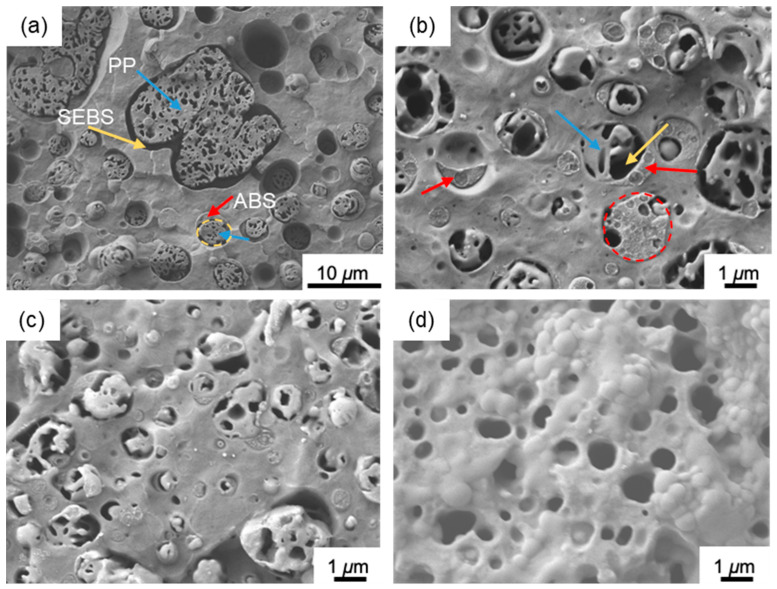
SEM micrographs of morphology of the PA6/(PP+*g*-POE)/ABS/SEBS (70/15/15/15) quaternary blends containing *g*-POE: (**a**) 0 wt%; (**b**) 5 wt%; (**c**) 10 wt%; (**d**) 15 wt%. All samples were cryofractured and then etched in *n*-heptane to remove SEBS. The PP, ABS and SEBS are indicated with blue, red and yellow arrows or circles, respectively.

Figure 2 show the SEM micrographs of the cryofractured surfaces of PA6/(PP+*g*-POE)/ABS/SEBS (70/15/15/15) quaternary blends with different contents of multi-phase compatibilizer POE-*g*-(MAH-*co*-St) at 0 wt%, 5 wt%,10 wt%, and 15 wt%, respectively. The SEBS phase is etched by *n*-heptane. For the blends without compatibilizer, as shown in Figure 2a, the morphology with large domain sizes about 15 μm was observed due to the poor adhesion between the interfaces. Due to the chemical affinity between PP and SEBS phases, a large porous dispersed phase is presented after etching the SEBS. Meanwhile, a thin shell, which is the ABS phase, can still be observed between the dispersed PP phase and the PA6 matrix. These morphology changes can be also predicted by the spreading coefficient equation as below [52]:

(1) $$\lambda_{A}=\gamma_{BC}-\left(\gamma_{AB}+\gamma_{AC}\right)$$

where $\gamma_{BC}$, $\gamma_{AB}$, and $\gamma_{AC}$ are the interfacial tension between each polymer pair, and the spreading coefficient $\lambda_{A}$ predicts the inclination of polymer A to autonomously disperse at the interface between B and C. If $\;\lambda_{A}>0$, then polymer A can spread at the interface, otherwise the spreading does not occur. Our previous work [46] has fully tested and demonstrated the effectiveness of the spreading coefficient.

As shown in Figure 2b, upon the addition of 5 wt% *g*-POE into the blends, the size of the structural domains was significantly reduced to about 2 μm with many smaller domains with a size of less than 1 μm dispersed in the PA6 matrix. With the content of *g*-POE increased to 10 wt%, the size of dispersed phase is further decreased and more smaller domains with a size under 1 μm are buried in the PA6 matrix, indicating the good compatibility between the components.

In addition, it can be observed that when there is no compatibilizer, the gap between the PP phase and the matrix is larger in Figure 2a, indicating that the etched SEBS phase is mainly distributed at the interface between the PP phase and the PA matrix. Due to the natural chemical affinity between PP and POE, as well as the excellent reactive compatibility between POE and PA6 matrix, as the *g*-POE content increases, the PP phase gradually migrates to the interface with PA6 matrix, while the SEBS phase migrates inward. At the same time, it is notable that the interface between the PP and PA6 becomes smoother, indicating that the compatibilizer *g*-POE successfully integrated together the incompatible PA6 and PP at their interface. In Figure 2b,c, a large number of rough cross-sectional spherical ABS phases can still be observed, which is caused by its partial etching with *n*-heptane. The ABS phase is more closely associated with SEBS or dispersed individually in the matrix, implying that the compatibilization effect of *g*-POE on ABS is limited and mainly used for compatibilization of the PP phase.

**Figure 3:**
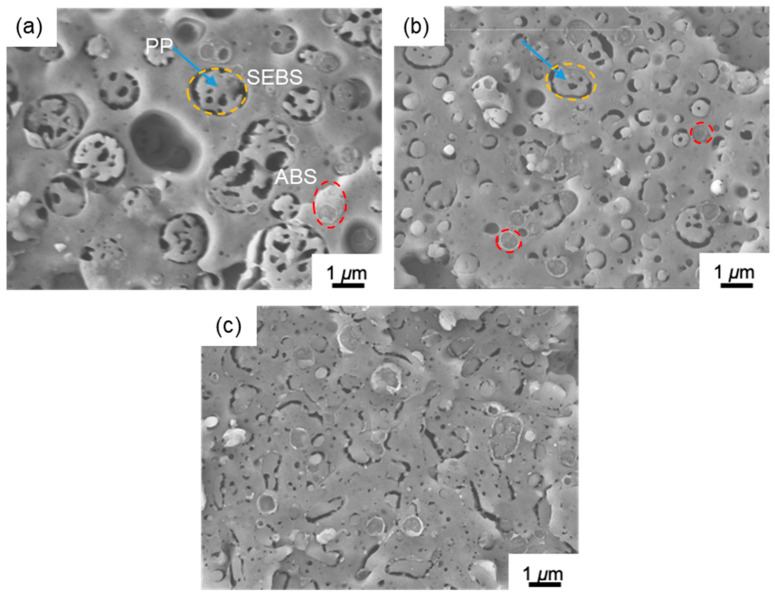
SEM micrographs of morphology of the PA6/PP/ABS/(SEBS+*g*-SEBS) (70/15/15/15) quaternary blends containing *g*-SEBS: (**a**) 5 wt%; (**b**) 10 wt%; (**c**) 15 wt%. All samples were cryofractured and then etched in *n*-heptane to remove SEBS. The PP, ABS and SEBS are indicated with blue, red and yellow arrows or circles, respectively.

Similarly, after etching with *n*-heptane, the SEM observation results of the PA6/PP/ABS/SEBS quaternary blend with *g*-SEBS as compatibilizer are shown in Figure 3. The size of the dispersed phases with the same morphology has been significantly reduced. Unlike *g*-POE, the etched SEBS is still mainly distributed at the interface between PP and PA6, with few dispersed inside the PP phase, indicating the presence of a hard shell-soft core structure with SEBS encapsulating PP. This is also due to the efficient reactive compatibility between *g*-SEBS and PA6, which promotes the migration of SEBS from the interior of the PP phase to the interface.

Based on mentioned above, both *g*-POE and *g*-SEBS have the ability to compatibilize the PA6/PP/ABS/SEBS quaternary blends, resulting in distinct and refined morphologies in the blends.

### 3.3. Mechanical Properties

**Figure 4:**
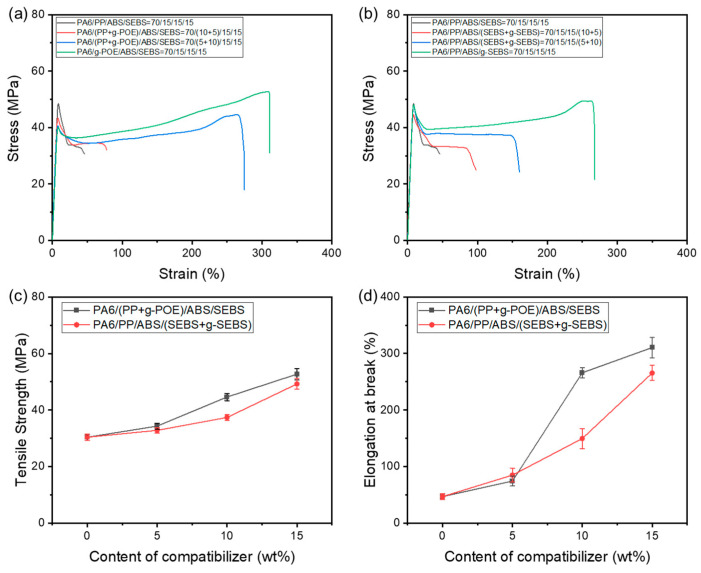
(**a**) Stress-strain curves of PA6/(PP+*g*-POE)/ABS/SEBS; (**b**) stress-strain of PA6/PP/ABS/(SEBS+*g*-SEBS); (**c**) tensile strength and (**d**) elongation at break of PA6/(PP+*g*-POE)/ABS/SEBS and PA6/PP/ABS/(SEBS+*g*-SEBS).

**Figure 5:**
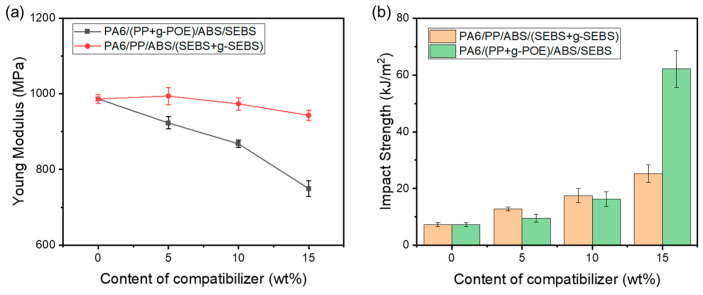
(**a**) Young modulus and (**b**) impact strength of PA6/(PP+*g*-POE)/ABS/SEBS and PA6/PP/ABS/(SEBS+*g*-SEBS).

The tensile curves and impact strength of the PA6/(PP+*g*-POE)/ABS/SEBS and PA6/PP/ABS/(SEBS+*g*-SEBS) blends are presented in Figure 4 and Figure 5. With increasing the content of the multi-phase compatibilizer *g*-POE or *g*-SEBS, both of them exhibit higher tensile strength and elongation at break. Compared to the PA6/PP/ABS/SEBS blends without compatibilizer, when *g*-POE reaches 15 wt% for PA6/(PP+*g*-POE)/ABS/SEBS blend, the elongation at break, tensile strength, and impact strength are increased by 563%, 73%, and 754%, respectively. When *g*-SEBS reaches 15 wt% for PA6/PP/ABS/(SEBS+*g*-SEBS) blend, they are increased by 467%, 62%, and 248%, respectively. This is mainly due to the excellent compatibilization effect of the multi-phase compatibilizer on the dispersed phase significantly reducing the dispersion size and increasing the interfacial quantity.

It should be noted that in Figure 5a the Young modulus of PA6/(PP+*g*-POE)/ABS/SEBS blend significantly decreases while PA6/PP/ABS/(SEBS+*g*-SEBS) blend only slightly decreases. The yield strength shows similar change with modulus in Figure 4a,b. The Young modulus reflects the material properties under low tensile elongation, when the material mainly undergoes affine transformation, so the modulus of blends mainly depends on the weighted average of each component. Compared to PP, POE has a lower degree of crystallization and exhibits elastomeric properties. Therefore, the addition of *g*-POE increases the overall content of elastomeric phase in the blend. With the increase in the low modulus POE elastomer replacing high modulus PP, Young modulus of blend decreases, resulting the same decrease in yield strength. In the PA6/PP/ABS/(SEBS+*g*-SEBS) blend, the replacement of SEBS with the multi-phase compatibilizer *g*-SEBS does not change the proportion of elastomer content, thus exhibiting more consistent modulus and yield strength.

Furthermore, *g*-POE and *g*-SEBS compatibilize the PA6 matrix and the dispersed phases of PP and ABS at the interfaces, forming a soft shell-hard core structure as observed in the morphology analysis above. The enhancement of interface strength makes it less likely for the blend to experience interface fracture under large deformation. In addition, when existing cracks encounter the dispersed phases with soft shell of *g*-POE or *g*-SEBS during expansion, the POE and SEBS elastomers can undergo significant reversible deformation immediately to prevent crack propagation, dissipating a large amount of energy, significantly toughening the blends [47]. Therefore, the elongation at break, fracture strength, and impact strength of the blend all increase with the increase in the content of compatibilizer. Concurrently, in the case of elastomer *g*-POE replacing the crystalline polymer PP, the total elastomer content increases and making the toughening effect of the elastomer more significant. When the *g*-POE content reaches 15 wt%, the impact strength increases significantly, achieving brittle-ductile transition.

### 3.4. Combination of g-POE and g-SEBS

**Figure 6:**
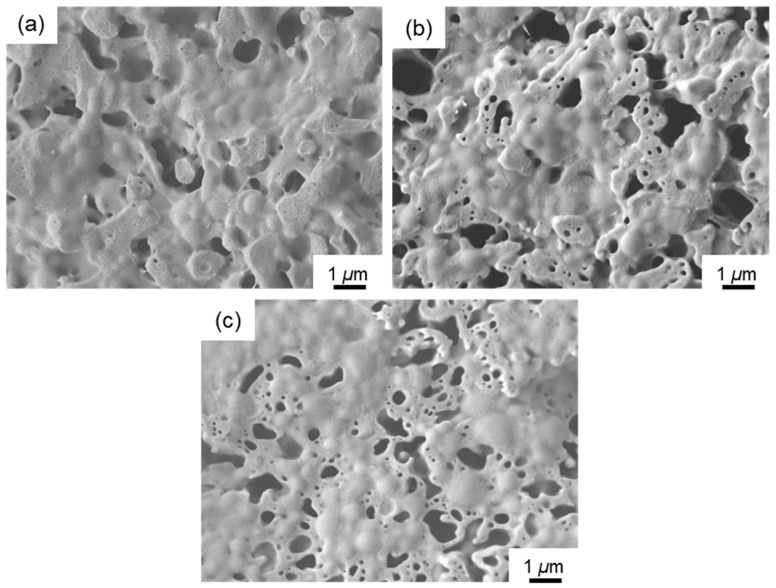
SEM micrographs of morphology of the PA6/(PP+*g*-POE)/ABS/(SEBS+*g*-SEBS) (70/15/15/15) quaternary blends containing *g*-POE and *g*-SEBS: (**a**) 5 wt% + 5 wt%; (**b**) 10 wt% + 10 wt%; (**c**) 15 wt% + 15 wt%. All samples were cryofractured and then etched in *n*-heptane to remove SEBS.

Furthermore, both *g*-POE and *g*-SEBS compatibilizers are added together in the quaternary blend to investigate their synergistic effect. Interestingly, after the same etching process, they exhibited distinct and complex phase morphologies compared to the when each compatibilizer is added respectively, as shown in Figure 6. When 10 wt% or above of each compatibilizer was added at the same time, the sea-island structure gradually disappeared, and the phase interface blended with each other instead. Near these interfaces, there are a large number of SEBS dispersed phases with small sizes of only 100–200 nm, which contribute to the larger interfacial area. This also implies that SEBS/*g*-SEBS and PP/*g*-POE exhibit gradient distribution near the interface. Due to the high reactivity of PA6 with *g*-SEBS and *g*-POE, as well as the good chemical affinity between SEBS and POE, they can be dispersed in each other to form a composite interface layer, distributed between PA6 and other dispersed phases. The composite interface layer has a higher distribution of *g*-SEBS near the SEBS phase or ABS phase and a higher distribution of *g*-POE near the PP phase. The formation of the gradient-changing composite interface layer effectively reduces its interfacial tension [53], allowing partial fusion of adjacent dispersed phases, showing a locally continuous morphology. At the same time, the gradient-changing composite interface layer can form a thicker effective interface thickness, providing stronger interface strength.

**Figure 7:**
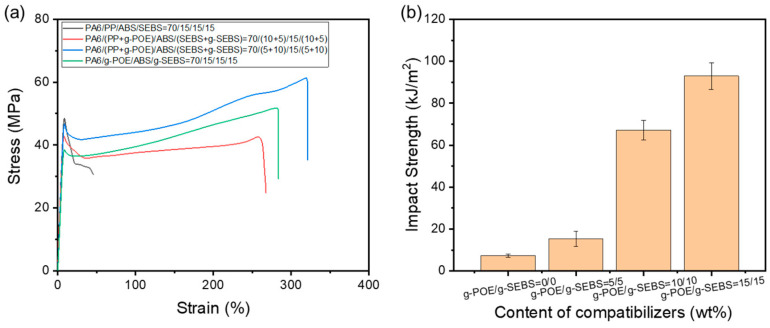
(**a**) Stress-strain curves, and (**b**) impact strength of PA6/(PP+*g*-POE)/ABS/(SEBS+*g*-SEBS).

The stress strain curves and impact strength of PA6/(PP+*g*-POE)/ABS/(SEBS+*g*-SEBS) quaternary blends are shown Figure 7 and Table 1. When both *g*-POE and *g*-SEBS compatibilizers are added together in PA6/PP/ABS/SEBS blends, it is found that the yield strain slightly decreased from 48.45 MPa to 43.51 MPa when each compatibilizer is added at 5 wt%, which is mainly because the increasing content of POE elastomer leads to the blend becoming softer. When both compatibilizers increased to 10 wt%, the yield strength instead increased to 47.18 MPa. The stress at break, elongation at break, and impact strength reached 62.61 MPa, 323.03%, and 67.20 kJ/m^2^. Compared to the PA6/PP/ABS/SEBS blend without compatibilizer, they were increased by 106%, 593%, and 823%, respectively, achieving a state of “super composites”. This is attributed to the formation of the composite interface layer when both compatibilizer were at 10 wt%, which is observed in Figure 6b, and provided stronger interfacial strength and more interface area. When the content of *g*-POE and *g*-SEBS continues to increase to 15 wt%, at this point the blends showed similar morphology with slightly decreased phase size, indicating that the enhancing effect of the compatibilizer has reached saturation. Therefore, the softening effect brought by the increase in the elastomer content once again takes the lead, which causes the yield strength and tensile strength to decrease again, but the impact strength continues increasing to 92.98 kJ/m^2^.

In PA6/PP/ABS/(SEBS+*g*-SEBS) quaternary blends, the impact strength basically increases linearly with the increase in *g*-SEBS content. The total elastomer content composed of SEBS and *g*-SEBS content remains at 15 wt%, a small increase in impact strength mainly stemming from the enhanced compatibility. However, in the PA6/(PP+*g*-POE)/ABS/SEBS blend, when the *g*-POE content increases from 10 wt% to 15 wt%, the impact strength undergoes a multiple-fold increase, showing a significant brittle-ductile transition. This is because with the increase in the amount of elastomer *g*-POE replacing the crystalline polymer PP, the total elastomer content composed of POE and SEBS/*g*-SEBS increases and triggers a brittle-ductile transition when total elastomer content exceeds 25 wt%.

It should be noted that when both compatibilizers are added simultaneously, the content of *g*-POE increases from 5 wt% to 10 wt%, and the brittle-tough transition occurs when the total elastomer content exceeds 20 wt%, indicating that the synergistic effect of increased compatibility and toughening by the elastomer compatibilizers promotes brittle-ductile transition at lower elastomer content.

**Figure 8:**
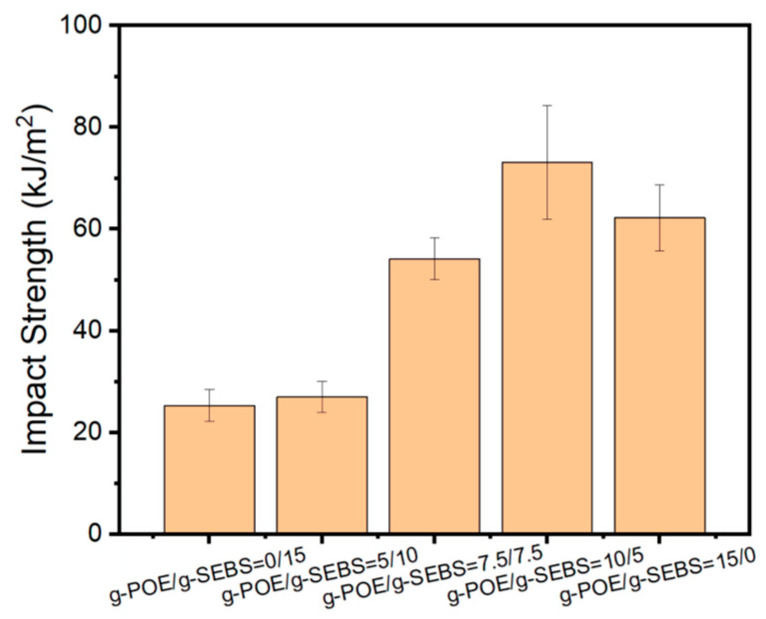
Impact strength of PA6/(PP+*g*-POE)/ABS/(SEBS+*g*-SEBS) blends with total 15 wt% compatibilizer content.

**Figure 9:**
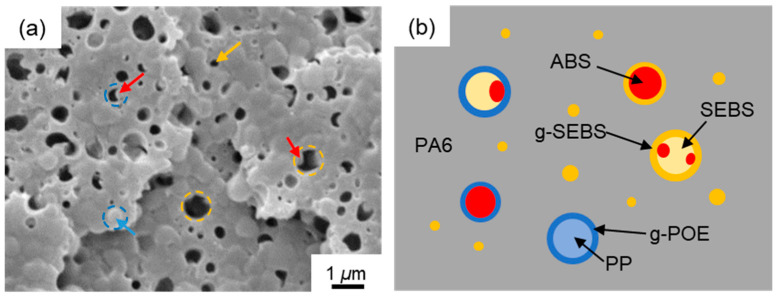
(**a**) SEM micrographs, and (**b**) schematic diagram of morphology of PA6/(PP+*g*-POE)/ABS/(SEBS+*g*-SEBS) (70/(5+10)/15/(10+5)). The sample was cryofractured and then etched in *n*-heptane to remove SEBS.

Further, we fixed the total content of compatibilizer content at 15 wt% and the relative content of *g*-POE and *g*-SEBS were adjusted to investigate their impact on the blend’s impact strength, as depicted in Figure 8. As the *g*-POE content increases to replace PP in the blends, the total elastomer compatibilizer content rises, leading to a significant enhancement in impact strength. It is noteworthy that when both compatibilizers are added simultaneously with a *g*-POE/*g*-SEBS weight ratio of 10/5, the blend exhibits a higher impact strength compared to when a single compatibilizer is added alone. The SEM image of PA6/(PP+*g*-POE)/ABS/(SEBS+*g*-SEBS) (70/(5+10)/15/(10+5)) is further analyzed, shown in Figure 9a. Compared with the single *g*-POE compatibilizer PA6/PP/ABS/*g*-SEBS (70/15/15/15) in Figure 2d, PA6/(PP+*g*-POE)/ABS/(SEBS+*g*-SEBS) (70/(5+10)/15/(10+5)) shows a more complicated morphology, of which a schematic diagram is shown in Figure 9b. Due to the reactivity between *g*-SEBS and PA6, SEBS could be individually dispersed in the matrix in sizes of hundreds of nanometers, resulting in a significant increase in interface area. Furthermore, the good affinity of SEBS with ABS and polyolefin facilitated the envelopment of the ABS phase by the SEBS phase, which was then dispersed in the matrix by *g*-POE, forming a three-layer encapsulation structure indicated by the red circles in Figure 9a. The combined use of the two compatibilizers demonstrates the synergistic effect of the enhanced compatibility and elastomeric toughening effect, thereby improving the performance of the polymer blend.

## 4. Conclusions

In summary, due to the high reactivity between multi-phase compatibilizer POE-*g*-(MAH-*co*-St)/SEBS-*g*-(MAH-*co*-St) and PA6, as well as the chemical affinity between POE-*g*-(MAH-*co*-St)/SEBS-*g*-(MAH-*co*-St) and PP, PS, and ABS, both exhibit strong compatibilization effects in the PA6/PP/ABS/SEBS quaternary blend. This is reflected in the significant reduction in dispersed phase size and the improvement in tensile and impact properties. As the proportion of the elastomer *g*-POE replacing the crystalline polymer PP increases, the total content of elastomer in the blend increases. The soft elastomer phase causes a significant decrease in the modulus and yield strength of the blends. However, it also exhibits an excellent toughening effect, especially when 15 wt% *g*-POE is added, where the blends show brittle-tough transition. When both compatibilizers are simultaneously added, it demonstrates superior compatibility with the dispersed phases through the formation of hierarchical interfaces. These hierarchical interfaces increase interfacial area and provide gradually changing interfacial strength, which can further dissipate more energies under deformation without sacrificing the yield strength. The fracture strength, fracture elongation, and impact toughness have increased by 106%, 593%, and 823% respectively, achieving remarkable and simultaneous growth in strength and toughness, leading to the realization of super composites. Consequently, this research presents a highly effective strategy for enhancing and toughening complex multi-component polymer blends, aiming to creating high-performance polymer blends and promoting the recycling of waste plastics.

## Figures and Tables

**Table 1:** Mechanical properties of PA6/(PP+*g*-POE)/ABS/(SEBS+*g*-SEBS) quaternary blends.

Content of *g*-POE and *g*-SEBS	Yield Strain (MPa)	Stress at Break (MPa)	Elongation at Break (%)	Impact Strength (kJ/m^2^)
0 + 0	48.45 ± 1.26	30.38 ± 0.87	46.8 ± 2.2	7.28 ± 0.75
5 + 5	43.51 ± 0.97	43.10 ± 1.23	256.6 ± 5.3	15.34 ± 3.56
10 + 10	47.18 ± 0.67	62.61 ± 2.56	324.8 ± 8.6	67.20 ± 4.61
15 + 15	39.09 ± 1.05	52.82 ± 1.42	290.6 ± 6.3	92.98 ± 6.39

## Data Availability

The original contributions presented in the study are included in the article, further inquiries can be directed to the corresponding author.
